# Exploring Water-Soluble South African *Tulbaghia violacea* Harv Extract as a Therapeutic Approach for Triple-Negative Breast Cancer Metastasis

**DOI:** 10.3390/cimb46100642

**Published:** 2024-09-26

**Authors:** Mohammed Alaouna, Rodney Hull, Thulo Molefi, Richard Khanyile, Langanani Mbodi, Thifhelimbilu Emmanuel Luvhengo, Nkhensani Chauke-Malinga, Boitumelo Phakathi, Clement Penny, Zodwa Dlamini

**Affiliations:** 1 Department of Internal Medicine, Faculty of Health Sciences, University of the Witwatersrand, Johannesburg 2193, South Africa; ala3570@gmail.com (M.A.); clement.penny@wits.ac.za (C.P.); 2 SAMRC Precision Oncology Research Unit (PORU), DSI/NRF SARChI Chair in Precision Oncology and Cancer Prevention (POCP), Pan African Cancer Research Institute (PACRI), University of Pretoria, Pretoria 0084, South Africa; rodney.hull@up.ac.za (R.H.); thulo.molefi@up.ac.za (T.M.); richard.khanyile@up.ac.za (R.K.); nkhens@icloud.com (N.C.-M.); 3 Department of Medical Oncology, Steve Biko Academic Hospital, University of Pretoria, Pretoria 0084, South Africa; 4 Gynaecologic Oncology Unit, Department of Obstetrics and Gynaecology, Charlotte Maxeke Johannesburg Academic Hospital, University of the Witwatersrand, Johannesburg 2193, South Africa; langanani.mbodi@wits.ac.za; 5 Department of Surgery, Charlotte Maxeke Johannesburg Academic Hospital, University of the Witwatersrand, Parktown, Johannesburg 2193, South Africa; thifhelimbilu.luvhengo@wits.ac.za; 6 Papillon Plastic Surgery, Suite 203B, 24 12th Avenue, Linksfield West, Johannesburg 2192, South Africa; 7 Department of Surgery, Faculty of Health Sciences, University of Kwa-Zulu Natal, Durban 4041, South Africa; phakathib@ukzn.ac.za

**Keywords:** migration, invasion, adhesion, oxidation, vanillin, schizandrin, taurolidine, alpha-pinene

## Abstract

Triple-negative breast cancer (TNBC) accounts for approximately 20% of all breast cancer cases and is characterized by a lack of estrogen, progesterone, and human epidermal growth factor 2 receptors. Current targeted medicines have been unsuccessful due to this absence of hormone receptors. This study explored the efficacy of *Tulbaghia violacea*, a South African medicinal plant, for the treatment of TNBC metastasis. Extracts from *T. violacea* leaves were prepared using water and methanol. However, only the water-soluble extract showed anti-cancer activity and the effects of this water-soluble extract on cell adhesion, invasion, and migration, and its antioxidant activity were assessed using MCF-10A and MDA-MB-231 cells. The *T. violacea* extract that was soluble in water effectively decreased the movement and penetration of MDA-MB-231 cells through the basement membrane in scratch and invasion tests, while enhancing their attachment to a substance resembling an extracellular matrix. The sample showed mild-to-low antioxidant activity in the antioxidant assy. Nuclear magnetic resonance spectroscopy revealed 61 chemical components in the water-soluble extract, including DDMP, 1,2,4-triazine-3,5(2H,4H)-dione, vanillin, schisandrin, taurolidine, and α-pinene, which are known to have anti-cancer properties. An in-depth examination of the transcriptome showed alterations in genes linked to angiogenesis, metastasis, and proliferation post-treatment, with reduced activity in growth receptor signaling, angiogenesis, and cancer-related pathways, such as the Wnt, Notch, and PI3K pathways. These results indicate that *T. violacea* may be a beneficial source of lead chemicals for the development of potential therapeutic medicines that target TNBC metastasis. Additional studies are required to identify the precise bioactive chemical components responsible for the observed anti-cancer effects.

## 1. Introduction

The subtype of breast cancer with the highest risk of spreading and the lowest survival rate is triple-negative breast cancer (TNBC) [1]. TNBC is the most aggressive form of breast cancer. In most countries and populations, ten to twenty percent of all breast cancers are TNBC cases [2] and as such, in the last 5 years, there has been an average of 170,000 TNBC cases per year worldwide [3]. TNBC is characterized by cellular mutations that lead to downregulated or absent expression of receptors for progesterone, estrogen, and human epidermal growth factor receptor 2 [4]. The absence of these receptors makes TNBC difficult to treat, since it is resistant to immune and hormone therapies [5]. Unlike other subtypes of breast cancer, the risk factors for TNBC include lifelong sensitivity to testosterone, shorter periods of breastfeeding, and giving birth to their first child at an older age [6]. TNBC is also found in younger patients diagnosed before the age of 39 [7]. Ethnicity is also an important risk factor for TNBC. People of African descent have a lower overall breast cancer risk than women of European descent, but they have higher mortality rates [8], with a higher incidence of TNBC. Additionally, black women tend to be diagnosed with TNBC at a younger age [9].

Genetic alterations commonly associated with TNBC include mutations in the *TP53* gene, which occurs in approximately 80–85% of patients [10]. *TP53* mutations are commonly associated with a lack of PR, ER, or HER2 expression [11]. Other genes that are commonly mutated in TNBC include RB1, BRCA1 and 2, PTEN, PIK3CA, USH2A, and MCL1; MYC expression is also increased in TNBC as is CCNE1 and FGFR2 expression [12].

Patients with TNBC are treated with non-specific cytotoxic multi-agent chemotherapy. The effectiveness of these treatments has been clinically demonstrated [13]. Despite their poor prognosis, TNBC tumors are particularly chemosensitive, albeit for a shorter duration than other breast cancers. Polychemotherapy has been shown to be effective in several trials; for example, taxane-containing regimens have demonstrated favorable effects on various disease-associated factors such as risk of recurrence, disease-free survival (DFS), and overall survival (OS) in TNBC [14].

Although antioxidant activity can prevent the development of cancer, antioxidants can promote tumorigenesis. One of the characteristics of cancer is an imbalance between ROS and antioxidants, which is normally defined as increased oxidative stress [15]. Cells that detach from the extracellular matrix (ECM) undergo apoptosis [16]. Apoptosis occurs through canonical apoptotic signaling and elevated ROS signaling pathways [17]. This means that any antioxidant process or compound that protects cells from ROS could also protect the cells from ROS-induced cell death. The survival of cells that detach from the ECM is promoted by antioxidant activity. This antioxidant activity can also promote cellular processes that promote tumor metastasis [18]. Small molecules that act as antioxidants, such as NAC (N-acetyl cysteine) and vitamin E, can reduce ROS levels and accelerate tumor metastasis [19].

Plants contain non-nutritive, bioactive, and diverse groups of organic chemicals known as phytochemicals [20]. Recently, phytochemicals isolated from green plants that have previously been used for medicinal purposes have become the focus of the search for new cancer-preventative and cancer therapeutic compounds [21]. Surprisingly, plants are the source of 47% of the FDA-approved drugs that can be used to treat cancer. Some of the recently synthesized anti-cancer compounds based on compounds isolated from plants include vinblastine and vincristine. These two anti-cancer drugs are derived from medicinal herbs such as comfrey [22]. Although several of these plant extracts have been explored as anti-cancer drugs in clinical trials, only a few affect the biochemical and molecular pathways that are actively involved in cancer/tumor formation and regulation, including cell cycle inhibitors, mitogenic signaling antagonists (to inhibit growth and proliferation), metastasis inhibitors, and immune system receptors [23]. One of the most common modes of action of phytochemicals in the treatment of cancer is the induction or modulation of autophagy and apoptosis [24]. In addition, phytochemicals may have antioxidant or pro-oxidant properties. Reactive oxygen species (ROS) are known to act as secondary messengers in a number of signaling cascades, including those directly related to cell proliferation. In this respect, ROS can be considered an important factor involved in the maintenance of cellular homeostasis. A mild increase in the generation of ROS, such as superoxide and hydrogen peroxide, has been shown to stimulate cell proliferation in several different cell types and may play a role in the carcinogenic process [25].

*Tulbaghia violacea* Harv. (Amaryllidaceae) is a small, bulbous plant native to South Africa. The plant is exclusively found in South Africa, namely, in the provinces of Natal, Gauteng, Northwest, Limpopo, Mpumalanga, and the Eastern Cape. The leaves are hairless and grow on slender, fleshy stems [26]. *T. violacea* (wild garlic) infusions in water have been used in traditional medicine in Southern Africa to cure a variety of ailments, including treating the symptoms associated with a variety of cancers. Many previous studies using this plant have indicated that it has verifiable effects on tumor cells.

Although *T. violacea* has been used in traditional medicine to treat various ailments, little scientific research has been conducted to validate its use. Previously *T. violacea* extracts using organic solvents have been shown to induce apoptosis in various cancer cell lines by inducing the overexpression of p53 [27,28,29]. It has also been shown that water-soluble extracts also induced apoptosis in cancer cells through increased expression of caspase 3 as well as through increased levels of ROS. The extracts were also shown to be selective for cancer cells, inducing cell death in these cells at a higher rate than in normal cells [30].

This study involved obtaining water- and methanol-soluble extracts from the leaves of *T. violacea* and testing their ability to inhibit the metastatic ability of triple-negative breast cancer cells by monitoring the effect of the extracts on cell adhesion, migration, and invasion. The antioxidant activity of the extracts was also measured using a DPPH antioxidant assay because of the link between antioxidant activity and the promotion of metastasis. Previously, IC_50_ values for these crude extracts were established using various cytotoxicity assays, which allowed us to test the effect of these extracts on the metastatic ability of a TNBC and normal breast cell lines at concentrations just below the IC_50_ as well at concentrations well below this value. In addition, the control breast cells were treated with the same concentration of the extract as the TNBC cells in further tests of the anti-cancer nature of these extracts.

## 2. Materials and Methods

### 2.1. Preparation of Plant Extracts

*T. violacea* plant extracts were prepared using aqueous extraction to obtain water-soluble extracts and methanol-based extraction to obtain non-water-soluble extracts. The species of the collected plants were confirmed by the staff at the C E Moss herbarium at the University of the Witwatersrand. The leaves of the plant were collected, rinsed with water, and cut into smaller pieces and dried for 120 h in a well-ventilated oven (Gallenkamp Genlab prime, Cambridges, UK) at 40 °C to ensure successful extraction. Once dried, the plant material was finely ground using an herb grinder (Fesh-Fesh) and passed through an 850-micron sieve. To extract water-soluble compounds, the dry powder was dissolved in 1 L of boiled water and allowed to cool for 24 h before filtering to obtain an aqueous extract. The filtrate was freeze-dried (Virtis Wizard 2.0, Warminster, PA, USA) to yield a dried aqueous plant extract, which was stored for 72 h.

In order to prepare a methanol extract containing compounds that are insoluble in water, the dried powder was dissolved in 250 mL of pure methanol. The filtrate was then placed in a Soxhlet extractor for 72 h. The crude methanol extract was then completely evaporated. The resulting powder was freeze-dried to form a dry powder.

Fresh solutions were prepared every day. For the aqueous extract, this involved taking 10 mg of powdered extract and dissolving it in either physiological saline, deionized water, or cell culture media, depending on the application. For the methanol extract, 10 mg of dried powdered extract was dissolved in a small amount of physiological saline, deionized water, or culture media; 25 μL of DMSO was added to all these solvents, giving a final concentration of 2.5% DMSO. Once the powder was dissolved, the volume was increased by adding 5 mL of physiological saline. This resulted in a final DMSO concentration of 0.5%. The diluted extract was applied to the cells for 24 h in fresh culture medium containing 10% FBS. Based on the high IC_50_ values for the methanol extract (820 μg/mL) and its inconsistent performance due to poor solubility, it was excluded from many metastasis assays and was only included in the antioxidant assay.

### 2.2. Cell Culture

MCF-10A (a non-tumorigenic breast epithelial cell line) and MDA-MB-231 (epithelial-like cells from a triple-negative breast cancer tumor) cell lines were used in this study. The cell lines were grown in culture flasks in DMEM media (Lonza, Basel, Switzerland, cat# BE15-604K) with penicillin/streptomycin (10,000 IU/mL) (Lonza, Basel, Switzerland, cat# 17-602E) and 10% FCS (Lonza, Basel, Switzerland cat#S711-001s), and incubated at 37 °C without CO_2_.

### 2.3. Measuring the Effect of Active Plant Extract on Cell Adhesion

To determine the effect of the water-soluble extract on the ability of MDA-MB-231 and MCF-10A cells to adhere to the ECM, Geltrex™ (Thermo Fisher, Waltham, MA, USA, cat#A1413201, Invitrogen), an extracellular matrix (ECM) analog, was used to simulate the ECM in the assays. The surface of 12-well plates (Corning cat#3460, Corning, NY, USA) was coated with 250 μL of a neat Geltrex™ solution. The plate was incubated for 30 min at 37 °C. The wells were washed with blocking buffer (0.5% BSA in DMEM F12 (Lonza cat# 12719F)) for 30 min. The cells were treated with the water-soluble extract at a concentration of 300 µg/mL, as previously described. After 24 h, the cells were washed and suspended in serum-free medium, and were then added to the wells at a concentration of 1 × 10^5^ cells/mL. The cells were incubated for 2 h, and the attached cells were washed and fixed for 10 min at room temperature. The cells were then washed and stained with crystal violet (Sigma-Aldrich, Burlington, MA, USA, cat# NC1635572) for 10 min. The dye was extracted from the stained cells using an SDS solution. Absorbance was measured at 550 nm (Spectramax multimodal plate reader). Control wells without cells were used as references. The absorbance was used as an indication of the number of cells that adhered to the ECM analogue.

### 2.4. Measuring the Effect the Active Plant Extract Had on Cell Invasion

A chemo-invasion assay was performed to determine the effect of the water-soluble extract on the ability of MDA-MB-231 and MCF-10A cells to invade other body tissues by penetrating an ECM analogue. Based on the original Boyden Assay [31], commercially available plastic inserts for multi-well plates, which possess a cell permeable membrane, as typified by Transwell^®^ Permeable Supports (Corning cat#3460), were used to perform accurate repeatable invasion assays. When placed in the well of a multi-well tissue culture plate, these inserts create a two-chamber system separated by a cell-permeable membrane. The cells were serum-starved for 24 h and then transferred to pre-prepared plates containing transwell inserts. These plates contained media with FBS that served as a chemoattractant in the well. The transwell inserts were 6.5 mm thick with 8 µm pores (Sigma cat Cls3422). The inserts were coated with neat Geltrex and allowed to set at 37 °C for 30 min. Approximately 1 × 10^6^ cells/mL were suspended in medium containing the *T. violacea* water-soluble extract at a concentration of 300 µg/mL, which was added to each insert. The cells were incubated at 37 °C for 24 h. The media was removed from the lower chamber, and the cells that had invaded into the lower chamber were fixed and stained with toluidine blue (Sigma cat#314) for 2 min. The dye was extracted from the cells using an SDS extraction solution for 1 h. The absorbance of the extracted dye at 620 nm was then measured using a SpectraMax M3 reader spectrophotometer (Molecular diagnostics, Sunnyvale, CA, USA).

### 2.5. Scratch Assay for Migration Analysis

A scratch/migration test was performed to investigate the effect of the plant extracts on the migration and proliferation rates of breast cancer and normal breast cells. The cells were grown in a cell culture plate until they formed a confluent cell monolayer. A cell-free zone was created by scoring the line through a confluent monolayer using a sterile pipette tip. The cells were then incubated with two concentrations of the *T. violacea* water-soluble plant extract, which were slightly lower than the IC_50_ (300 µg/mL) and half the IC_50_ (200 µg/mL). Cells were treated with 5FU were used as a positive control, while the other cells were left untreated. Cell migration and proliferation were monitored microscopically over a 48 h period. Measurements were taken every 2 h for the first 6 h, and then at the 24 and 48 h marks. Changes in the width of the gap (which decreased as the cells proliferated and migrated) indicated cell migration. The width of the scratch was plotted against time to analyze the effect of the extracts on the cells.

### 2.6. Antioxidant Assay

To determine whether the plant extracts had antioxidant activity, the 2,2-diphenyl-1-picrylhydrazyl (DPPH) assay was performed. The assay was performed according to the method described by Blois [32]. A standard solution of DPPH was prepared by dissolving the DPPH powder (Sigma-Aldrich, Burlington, MA, USA, Cat# 044150.03) in methanol to obtain a final concentration of 50 µg/mL. Various concentrations of the extracts were prepared based on the IC_50_ values. A 96-well plate was prepared with a methanol-only blank, a reference sample of the DPPH solution only, and then various concentrations of the extracts in triplicate with the DPPH solution added. The plate was then incubated for 30 min in the dark. The absorbance of the wells was measured at 515 nm using a plate reader to determine the number of neutralized radicals. The percentage of DPPH used was calculated using the following formula:

A515 of tested compound − blank

100% − A515 initial DPPH methanol solution − blank × 100

The concentration required to neutralize 50% of the DPPH (EC50) was determined by plotting the natural logarithm of the %DPPH remaining versus the concentration of the sample.

### 2.7. Determining the Molecular Composition of the Water-Soluble Extract

An NMR analysis was performed on the water-soluble extract of *T. violacea* to identify the chemical constituents of this crude extract. The NMR tube was cleaned, and approximately 10 mg of the starting material was added. All substances were dissolved before the samples were gently shaken. To ensure that all the samples were exposed to a uniform magnetic field, the spinner was turned after it was placed in the magnet. To eliminate fingerprints and grime, 2-propanol and laboratory tissues were used to wipe the exterior of the NMR tube. The rotor of an autosampler-equipped Varian 600 MHz spectrometer was employed. After the NMR test was completed, the spectra were analyzed, and peaks were assigned. The NMR spectrum was interpreted using an appropriate application to analyze the spectrum (MestReNova v14.3.3, from Mesytalab Research, Santiago de Compostela, Spain).

### 2.8. Next-Generation Sequencing to Measure the Transcript Levels of Genes Involved in Proliferation and Metastasis

Next-generation sequencing was performed by Inqaba Biotechnology Industries (Pretoria, South Africa). RNA fragments were analyzed using a bioanalyzer. A sequencing library was prepared using the MiSeq RNA-0 rRNA reduction library kit (Illumina, San Diego, CA, USA, cat# 20020492). Sequencing was performed using a NextSeq300 system (Illumina, San Diego, CA, USA. Paired-end sequencing was performed at a depth of 10 million bases over 300 cycles. Following sequencing, the raw sequencing data were analyzed using the Galaxy platform (Galaxy Europe ver 22.05 (galaxyproject.eu)) with a pipeline consisting of the following tools: Trimmomatic, which was used to trim the reads, and FastQC, which was used to generate the quality control reports. HiSAT2 was used to align the reads. Differential gene expression was analyzed using the Limma package ver 3.52.4 (limma-voom). The HG38 human genome was used as the reference genome, and reference mapping was performed using bowtie2. PANTHER was used to identify genes involved in invasion, metastasis, and adhesion. The feature counts for the transcript levels were compared between the treated and untreated cell extracts.

### 2.9. Statistical Analysis

When comparing the means for the data obtained from the adhesion, migration, and invasion assays, one-way ANOVA with the Bonferroni post hoc multiple comparisons test was performed. Tukey’s test was then used for pairwise mean comparisons. For the migration assay, due to the inconsistent response of the normal cell line to the initial scratching of the monolayer, we performed Levene’s test for homogeneity of variance. The significant differences between the treated and untreated samples were tested using one-way ANOVA with Bonferroni correction.

## 3. Results

### 3.1. Effect of the Extract on Cell Adhesion

**Figure 1:**
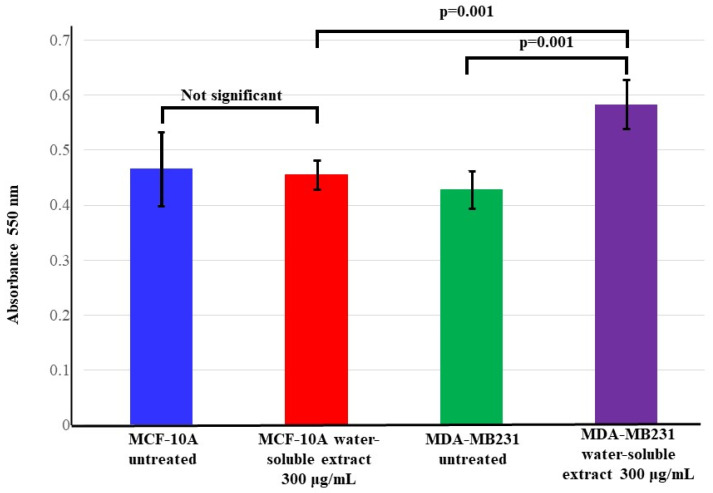
Effect of the extract on cell adhesion of TNBC and normal breast cell lines. The control MDA cells had the lowest number of cells that adhered to the surface of the matrix. This was significantly increased following treatment with the water-soluble *T. violacea* extract. Significant differences between the treated and untreated samples were found using one-way ANOVA with the Bonferroni post hoc multiple comparisons test.

To assess the effect of the *T. violacea* water-soluble leaf extract on cell adhesion in the triple-negative breast cancer cell line MDA-MB-231 and the normal breast cell line MCF-10, an adhesion assay was performed using Geltrex^TM^ as an ECM analog and the number of cells able to adhere to the surface before and after treatment was determined. The number of cells was determined using a dye-absorption assay. As shown in Figure 1, the extract increased the number of MDA-MB-231 cells that adhered to the ECM analog, with greater numbers of these cells being found on the matrix compared to the treated and untreated normal breast cells. At the same time, it was observed that the treatment had no effect on the number of normal cells that adhered to the ECM. As expected, the untreated MDA-MB-231 cells had fewer cells adhering to the synthetic ECM than the normal breast cells. This indicates that the *T. violacea* crude water-soluble extract was able to increase cell adhesion in a population of TNBC cells.

### 3.2. Effect of the Extract on Cell Invasion

**Figure 2:**
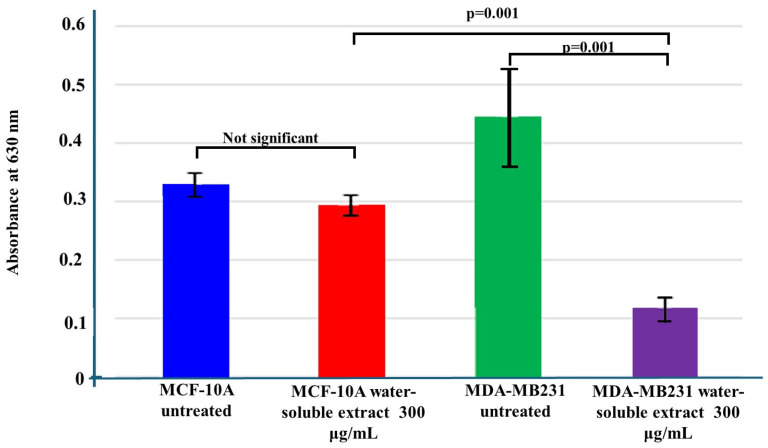
Effect of the extract on the invasive ability of TNBC and normal breast cell lines. The high absorbance for the untreated MDA-MB-231 samples indicates that many of these cells invaded through the ECM matrix, and so, the amount of dye absorbed was high. The treatment of these cells led to a large decrease in the number of invading cells. The extract had very little effect on the normal cells. The significant differences between the treated and untreated samples were tested using one-way ANOVA with the Bonferroni post hoc multiple comparisons test.

Since the ability of cells to penetrate and cross the ECM is vital for metastasis, an invasion assay was performed to test the effects of the water-soluble extract on the invasive properties of MDA-MB-231 and MCF-10A cells. Both cell lines were treated with concentrations of the extract just below the IC_50_, which was determined in a previous study. The IC_50_ value determined for MDA-MB-231 cells was approximately 395 µg/mL after 72 h, while the IC_50_ value determined for MCF-10A cells was 537 µg/mL. The selected concentration of 300 µg/mL was predicted to kill approximately 40% of cells, and treatment of the TNBC cell line, MDA-MB-231, with the *T. violacea* water-soluble extract significantly decreased the ability of MDA-MB-231 cells to penetrate and cross the ECM analog (*p* = 0.001). This implies that it was able to decrease the ability of these cells to invade other tissues. It had no significant effect on the ability of MCF-10A cells to invade through an ECM analog, as the treated and untreated cells showed no significant difference in the number of cells that invaded through the ECM (Figure 2). The innate ability of MCF10A cells to invade the ECM was significantly less than that of untreated MDA-MB-231 cells (*p* = 0.001). The invasive ability of this TNBC cell line decreased below that of normal breast cells following treatment.

### 3.3. Measuring the Effect of the Active Plant Extract on Cell Migration

**Figure 3:**
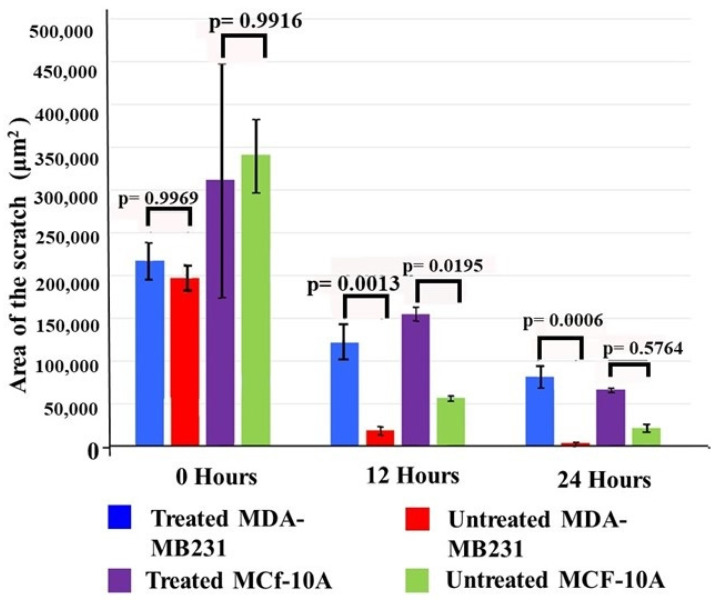
Effect of the extract on the migratory abilities of TNBC and normal breast cell lines. Changes in the scratch area following treatment with the extract were analyzed. Initially, there was no significant difference at 0 h, and no statistically significant difference was observed between the untreated and treated cells in either cell line. Treatment of either cell line with the extract for 12 h inhibited cell migration. This effect persisted in the MDA-MB-231 cells. However, the MCF-10A cells either recovered or were less affected by the extract. The significant differences between the treated and untreated samples were tested using one-way ANOVA with the Bonferroni post hoc multiple comparisons test. Due to the size of the error bars at 0 h, Levene’s test for homogeneity of variance was performed. For the MCF-10A samples at 0 h, the homogeneity of variance assumption was not met (F-ratio = 16.07997, *p*-value = 0.002478).

The wound-healing or scratch assay was one of the earliest methods developed to study cell migration in vitro [32]. This method is based on observations of cell migration into a “wound” that is created in a cell monolayer and to some extent mimics cell migration. This assay was performed to ascertain the potential effects of the crude extract on cell migration. Figure 3 displays the scratch area that was measured using Olympus EVOS M7000 imaging software version 1.0 and a light microscope (Olympus CKX41, Olympus, Hachioji, Tokyo, Japan) It can be seen from these plots that there was no significant difference in the area of the scratch between treated and untreated cells in either cell line (*p* = 0.9969 and *p* = 0.9916) at 0 h, indicating that the scratch size was the same between the treated and untreated cell lines. However, MCF10A cells showed large variances between replicates with large error bars for the standard error. Levene’s test for variance of homogeneity showed that the MCF10A cells at 0 h did not meet the requirement for homogeneity (f-ratio = 16.07997, *p*-value = 0.002478). This indicated that the normal cell line reacted differently to the physical act of scratching the cell monolayer. Therefore, there does seem to be a significant difference between the treated and untreated MCF-10 cells at 0 h. As such, a logarithmic adjustment to the data was performed and the ANOVA was repeated

After 12 h, there was nearly complete closure of the scratch in the untreated MDA-MB-231 cells, while the cells treated with *T. violacea* water-soluble extract showed a significantly larger cleared area where cell migration had not occurred (*p* = 0.0132). This lack of closure in the treated cells still persisted at the 24 h mark (*p* = 0.0006). A similar situation was observed in the normal MCF-10A breast cells, where there was a significant difference in the area of the scratch between treated and untreated cells at 12 h (*p* = 0.01953). However, after 24 h, there was no significant difference between treated and untreated MCF-10A cells (*p* = 0.5764). This indicates a weaker or shorter-lived effect of the extract on MCF-10A normal breast cells.

**Figure 4:**
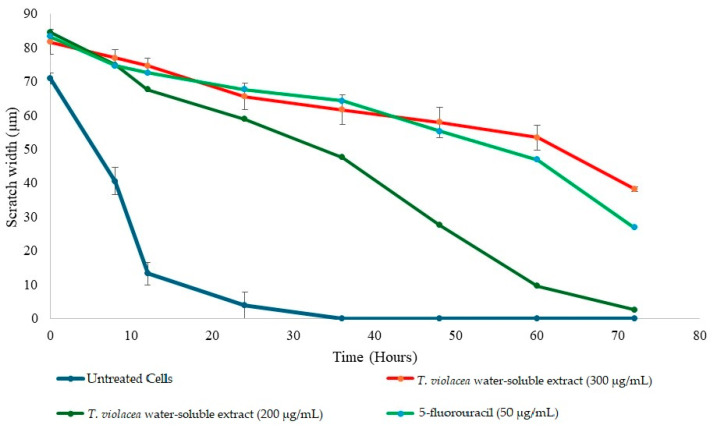
Effects of different concentrations of the water-soluble extract on the migratory abilities of TNBC cell lines. Changes in scratch width in μm^3^ over time following treatment with the extract. The untreated cells migrated rapidly and by the 60th hour after treatment, the scratch was fully closed in the untreated cell line. The scratch in cells treated with lower concentrations of the water-soluble extract closed more rapidly and to a greater degree than the scratch in the positive control and cells treated with higher concentrations of water.

The effect of the various concentrations of the extract on the narrowing of the scratch in TNBC cell lines is shown in Figure 4. The untreated cells rapidly migrated to close the leading edges of the scratch after approximately 35 h. The highest concentration of the extract was as effective as 5FU at inhibiting cell migration. This concentration was previously established to kill 40% of MDA-MB-231 cells. A concentration of 200 µg/mL was previously shown to kill approximately 20% of cells. The lowest concentration of the extract (one-third of the IC_50_ value) was previously shown to kill less than 5% of MDA-MB-231 cells, and only delayed the closure of the scratch by approximately 25 h.

### 3.4. Determination of Antioxidant Activity Using the 2,2-Diphenyl-1-picrylhydrazyl (DPPH) Radical-Scavenging Method

**Figure 5:**
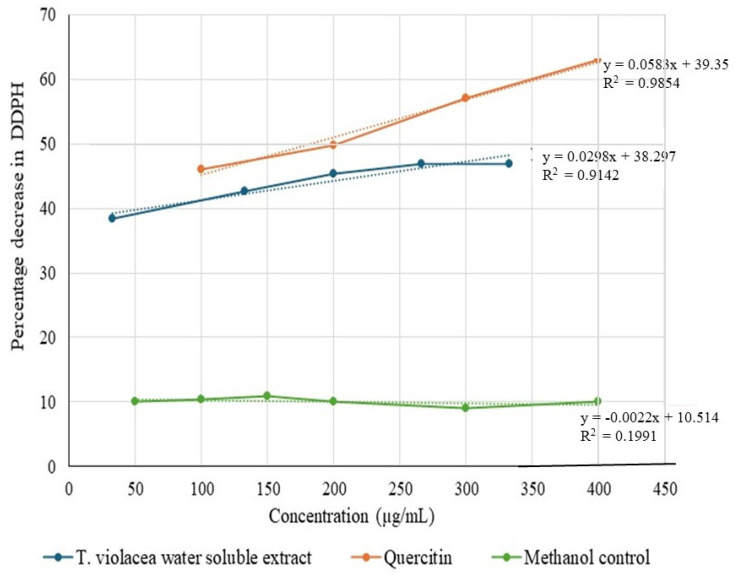
Antioxidant assay. The ability of the extract to scavenge DPPH free radicals is a reflection of its antioxidant activity. The activity of the water-soluble extract of *T. violacea* was similar to that of the positive control, quercetin. This implies that the water-soluble extract has antioxidant activity. The corresponding dotted trendlines give a clearer indication of the trend of antioxidant activity trend of each sample.

Antioxidant activity was measured in terms of the hydrogen-donating ability or the radical-scavenging ability of the extracts using the stable radical DPPH. The experiments were performed according to the method described by Blois [33]. The amount of sample necessary to decrease the absorbance of DPPH by 50% (IC50) was calculated graphically for the water-soluble solutions of *T. violacea* at different concentrations. Figure 5 shows the results of this assay. The water-soluble extract showed antioxidant activity with a Y-intercept similar to that of the positive control, quercetin. However, as the concentration of quercetin increased, the antioxidant activity increased rapidly, whereas an increase in the concentration of the extract resulted in only a small increase in antioxidant activity. The IC_50_ value for the water-soluble extract was calculated to be 393 μg/mL. This value is much higher than that of classic antioxidants, as well as most of the many plant extracts, as presented in Table 1, implying that the antioxidant activity of the extract is very low and, as such, does not promote metastasis.

### 3.5. Identification of Compounds Using NMR

**Figure 6:**
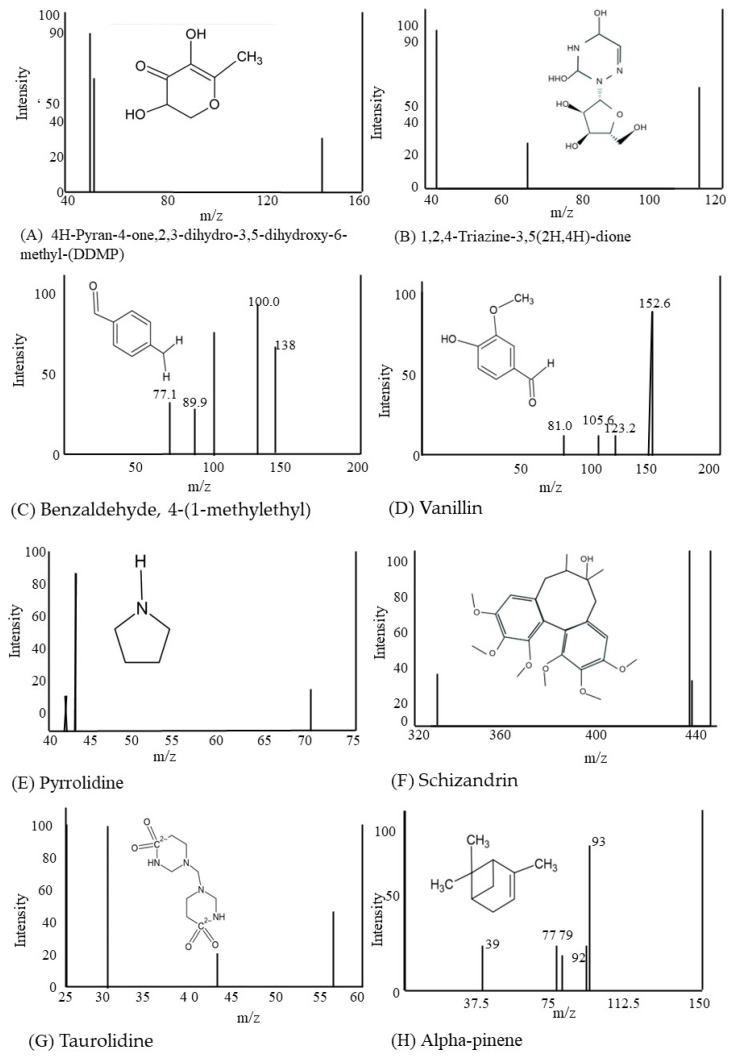
Compounds identified in the water-soluble extract with suspected antioxidant or anti-metastatic activity. Of the 61 compounds identified using NMR, these 8 compounds were identified as having either antioxidant activity or have published studies on their anti-metastatic activity.

An NMR analysis of the water-soluble extract indicated the presence of 61 compounds. A list of these compounds is provided in Table 2. The names of these compounds were used as queries to search PubChem in order to identify compounds with known anti-cancer activity. Eight compounds were identified that have known anti-cancer activity. These are 4H-pyran-4-one, 2,3-dihydro-3,5-dihydroxy-6-methyl-(DDMP), 1,2,4-triazine-3,5(2H,4H)-dione, benzaldehyde, 4-(1-methylethyl), vanillin, pyrrolidine, schizandrin, taurolidine, and alpha-pinene. Some of these compounds have known medical uses and applications, whereas others have known applications that are not related to medical applications. Finally, some of the identified compounds have no known uses. The structures of the eight compounds with known anti-cancer activity and their mass charge spectra are shown in Figure 6.

### 3.6. Transcription Profiles of Genes Involved in Adhesion, Invasion, and Metastasis

**Figure 7:**
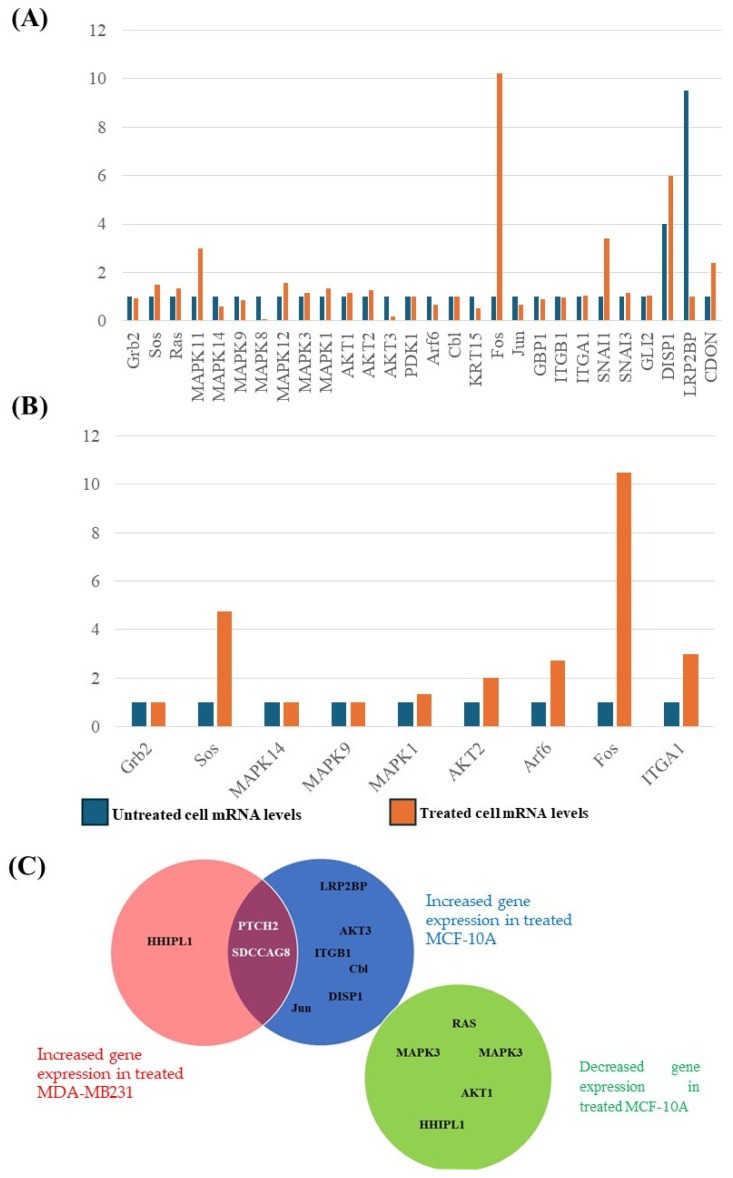
Levels of transcripts for genes involved in migration, invasion, and adhesion detected by NGS analysis of treated and untreated TNBC and normal breast cell lines. (**A**) Fold change in transcripts identified in MDA-MB-231 TNBC cells. The treatment resulted in an increase in the transcription of MAPK11, c-Fos, DISP1, and CDON. The levels of LRP2BP decreased following treatment. (**B**) The fold changes observed for these genes in MCF10A normal breast cells showed an increase in the transcription of Sos, AKT2, Arf6, c-Fos, and ITGA1. In both (**A**,**B**), red signifies the transcript level following treatment, while blue indicates the transcript level of untreated cells. (**C**) Genes whose transcription level was only detected after treatment in TNBC cells (red circle) or normal cells (blue circle). Additionally, the level of transcripts for some genes became undetectable in normal cells after treatment (green circle).

Next-generation sequencing of RNA extracted from both MDA-MB231 and MCF-10A cells before and after treatment with the water-soluble *T. violacea* extract allowed us to establish the levels of transcripts (feature counts) in the transcriptomes of these cells. Using PANTHER to classify all the genes identified in this analysis based on their biological roles, all the genes that were involved in invasion, adhesion, and metastasis were identified and their levels of transcription before and after treatment were compared. Figure 7 shows the results of this analysis, with Figure 7A depicting the fold change in transcripts identified in the MDA-MB-231 TNBC cell line, while 7B depicts the fold change observed for these genes in MCF10A normal breast cells. Figure 7C shows the genes whose transcription was detected only after treatment in TNBC cells (red circle) or normal cells (blue circle). Additionally, the levels of the transcripts for some genes became undetectable in normal cells after treatment (green circle).

SNAI1 and 3 negatively regulate cell adhesion [69]. The transcription levels of both of these genes were increased in MDA-MB-231 cells following treatment. The same was true for Gli2. The inhibition of Gli2 has been associated with decreased migration and invasion [70]. DISP1, HHIPL1, SDCCAG8, and PTCH2 are components of the sonic hedgehog pathway that plays a role in stimulating invasion and metastasis [71]. The transcription of both of these genes increased following treatment with the water-soluble extract. CDON mediates cell adhesion, and its transcription increased following treatment with the extract [72]. LRP2BP is involved in cell migration and metastasis and was downregulated following treatment. The Ras signaling pathway is involved in cell migration and the transcription of components of this pathway (Grb2, Ras, and Sos [73]) remained largely unchanged. Several mitogen-activated protein kinase (MAPK) transcripts were detected. MAPKs are known to play a role in cell migration [74]. The transcript levels of most of these MAPKs remained unchanged following treatment, except for MAPK11, which increased following treatment. Components of the AKT pathway also play a role in cell migration and invasion [75]. The transcript levels of these genes remained largely unchanged. c-Fos knockdown results in decreased migration, invasion, and metastasis [76]. The only genes that showed major increases in transcription following treatment were c-fos, MAPK11, SNAIL1, CDON, and DISP1. In the MCF10A cell line, the treatment resulted in the absence of multiple transcripts, including those for multiple MAPKs, and AKT and Ras pathway components.

## 4. Discussion

The ability to invade tissues and penetrate the extracellular matrix of basement membranes and stromal compartments is a major reason why metastasis is the principal cause of cancer-related deaths [77,78]. Inhibition of cancer migration and invasion is an attractive target for the development of new therapies. The ability of cancer cells to metastasize depends on their adherence, ability to invade other tissues, and ability to move and migrate. The effect of the water-soluble extract on the ability of MDA-MB-231 cells to metastasize was assessed using various assays.

### 4.1. Cell Invasion, Migration, and Adhesion Assays

Cell differentiation, cell cycle, migration, and survival can all be stimulated by cell adhesion [79]. It also plays an essential role in cell communication, regulation, development, and the maintenance of tissues. Changes in cell adhesion can be a defining event in cancer [79,80]. In cancer cells, adhesiveness is generally reduced due to lower intercellular adhesion, allowing cancer cells to dissociate from other cells [80]. Tumor cells are characterized by changes in their adhesion to the ECM, which may be related to their invasive and metastatic potential. The ability of MDA-MB-231 cells to adhere to a synthetic analog of laminin ECM components was evaluated following treatment with the water-soluble extract and assessed using a toluene blue dye absorption assay. The water-soluble extract decreased the migration ability of these cells by increasing their adhesion to the ECM. At the same time, the extract had no effect on normal breast cell adherence to the ECM.

In vitro invasion assays were performed to better understand the effect of the *T. violacea* crude extract on the metastasis process. Neoplastic cells require the ability to invade the surrounding tissue or enter the blood or lymphatic system after adhering to cell membranes in vivo. Once attached to a target basement membrane, they are enzymatically digested by type IV collagenase, allowing for entry into the circulatory system, migration, and finally the establishment of a metastatic (secondary) tumor by re-attachment of the migrating cells to the blood vessel [81]. Invasion assays, which mimic this process, provide an indication of the ability of cells to pass through an ECM (laminin-1)-coated membrane similar to the basal lamina. Untreated MDA-MB-231 cells migrated through the matrix-coated membrane and attached to the underside. In contrast, the inclusion of the *T. violacea* crude water extracts in the assay significantly hampered cell invasion, resulting in far fewer cells traversing the membrane. These findings are consistent with those of the adhesion assays, as cell adhesion is required for invasion. Previous studies using breast, lung, cervical, prostate, and colon cancer cell lines found a positive correlation between adhesion and invasion [82].

The effect of the extracts on the migration ability of MDA-MB-231 cells was assessed using scratch or wound assays. The scratch assays showed that the scratch area decreased rapidly when the cells were left untreated, which was also observed when the distance between the two cell fronts was used as an indication of wound closure. The *T. violacea* water-soluble extract showed consistent effects on the migration of MDA-MB-231 cells at a concentration lower than the IC_50_ at 300 μg/mL, which was previously shown to kill less than 40% of cells. This effect occurred in the first 12 h following treatment and persisted for 24 h until approximately 35 h after treatment, when the effect began to decrease and cell migration increased. Since no further extract was added, this time indicates the active lifespan of the components within the extract or how long the cells take to recover. Using half of the IC_50_ concentration of the extract, it was observed that the inhibitory effect on cell migration ended after only 24 h. However, even at this lower concentration, the wound reached full closure after 72 h. This seems to indicate that the cells took time (approximately 24 h) to recover from the inhibitory effects of the extract on cell migration and it was not due to the continued activity of the extract. This effect of the *T. violacea* water-soluble extract was not exclusively due to increased cell death and seems to be due to the effect of the extract on the ability of cells to migrate by targeting the molecules or signaling pathways involved in migration. The migration of normal MCF-10A breast cells was negatively affected by the extract. However, the effect was not as strong or as long-lasting as that on TNBC cells. As expected, normal cells migrated at a slower rate than cancer cells.

### 4.2. Assay for Antioxidant Activity

Plants are rich in secondary metabolites that are natural antioxidants and their antioxidant activity is frequently linked to the presence of phenolic compounds. Polyphenolic compounds are the most potent natural antioxidants among the various plant secondary metabolites [83,84].

The water-soluble extract of *T. violacea* demonstrated weak-to-moderate antioxidant activity, particularly in reducing DPPH radicals [85]. However, the role of antioxidants in cancer progression and metastasis is intricate, as excessive antioxidant activity may potentially protect cancer cells from oxidative stress-induced cell death, thereby promoting tumor growth and metastasis [86]. Therefore, it is crucial to carefully evaluate the antioxidant properties of the *T. violacea* extract in the context of its potential anti-metastatic effects [27]. A balanced approach is necessary to understand the interplay between the extract’s antioxidant and anti-cancer activities, as well as the underlying mechanisms involved [87]. Further research is warranted to elucidate the specific compounds responsible for these activities and their potential therapeutic applications [88] (Figure 5). The compounds that scavenge DPPH radicals are expected to be hydrophilic radical scavengers because of their presence in the water-soluble extract. One of the identified water-soluble radical scavengers was H-pyran-4-one, 2,3-dihydro-3,5-dihydroxy-6-methyl (DDMP). DDMP is a strong antioxidant [89] that can be formed non-enzymatically from hexose [90], and thermal degradation of D-glucose to form DDMP has been reported [91]. This study identified DDMP as a major water-soluble radical scavenger present in the *T. violacea* water-soluble extract. This may have contributed to the ability of the extract to act as a radical scavenger of DPPH radicals in the antioxidant assay. DDMP is known for its potent antioxidant properties [92]. The second relevant compound, pyrrolidine, is a nitroxide, a stable free radical with an unpaired electron in its nitroxyl group and therefore has antioxidant activity. Nitroxides can accept electrons from reactive oxygen species (ROS) and convert them into stable, non-radical forms. This process helps neutralize ROS and reduce oxidative stress, making nitroxides valuable as antioxidants and potentially useful in various medical and biological applications [48].

### 4.3. Effect of the Identified Compounds on Metastasis and Invasion

To achieve successful motility, cellular signaling networks are activated, which results in morphological changes [93]: cells lose their epithelial characteristics and adopt mesenchymal-like characteristics, such as E-cadherin expression. These signaling networks include the Wnt/β-catenin and Hh pathways [94]. Based on our findings, the *T. violacea* water crude extracts were able to disrupt the pathways involved in MDA-MB-231 migration and their ability to populate a cell-free zone. This implies that the crude *T. violacea* extracts are effective in promoting cell adhesion and impeding cell invasion and migration, thereby decreasing their metastatic ability.

Triazine-3,5(2H,4H)-dione (6-azauracil) is a pyrimidine analog that has demonstrated substantial antitumor effects against various transplantable mouse tumors [95]. The 1,2,4-triazine ring is a prominent structural motif in many naturally occurring and synthetically derived biologically active compounds. These include anti-cancer and anti-inflammatory agents [96]. Cytosine arabinoside is one of the three most important pyrimidine antimetabolites in cancer chemotherapy [97]. Well-established chemotherapeutic agents, such as 5-fluorouracil (5FU), 6-mercaptopurine (6MP), 6-thioguanine (6-TG), cytosine arabinoside (ARA-C), and methotrexate (MTX), inhibit cancer cell proliferation and survival by inhibiting DNA synthesis. These drugs target different aspects of cancer cell growth and replication, making them valuable tools for cancer chemotherapy [98]. Research performed on 6-azauridine (6AU) by the National Cancer Institute using animal models demonstrated that this compound led to hematological enhancements and could be used to treat newly diagnosed cancers [99]. The biological activities of azanucleosides, nucleoside analogs with a furanose ring replaced by a nitrogen-containing ring or chain, have been a subject of research interest [100]. 6AU is known to inhibit transcription by depleting the intracellular pool of guanosine monophosphate (GMP) and uridine monophosphate (UMP) [101].

Benzaldehyde, or 4-(1-methylethyl)-(cuminaldehyde), is known to cause lysosomal vacuolation, acidic compartment enlargement, cytotoxicity, and inhibition of topoisomerase I and II activities, thereby decreasing tumor size [102]. It is known as an agent capable of inhibiting cell growth [103].

Vanillin, chemically known as 4-hydroxy-3-methoxybenzaldehyde, has shown anti-cancer properties, mainly owing to its strong antimutagenic action [104]. The FDA considers vanillin safe for use in food and pharmaceutical products, due to its oral LD_50_ in rats ranging from 1.58 to 2.8 g/kg [105]. Vanillin has been demonstrated to decrease MMP-9, an enzyme responsible for extracellular matrix disintegration, which aids cancer cell invasion and metastasis. By inhibiting MMP-9, vanillin may further limit the invasive and metastatic potential of cancer cells via the downregulation of the nuclear factor-B (NF-κB) signaling pathway in human hepatocellular carcinoma cells [106]. The ability of vanillin to prevent cancer cell invasion and migration at non-lethal dosages may be due to its ability to inhibit DNA repair mechanisms. Vanillin also exhibits antioxidant activity. It inhibits DNA-dependent protein kinases, enhancing cancer cell sensitivity to cisplatin [107].

The structural similarities between vanillin and acetyl salicylic acid and their potential anti-invasive effects in different cancer cell lines call for further research into their therapeutic capabilities in impeding cancer cell invasiveness [107]. Previous studies have confirmed the anti-metastatic effectiveness of vanillin against breast cancer cells in laboratory and animal models [108]. Vanillin obstructs cell migration and the breakdown of the extracellular matrix (ECM), which is crucial for cancer invasion. This suggests that vanillin can effectively prevent cancer invasion in experimental models and may help reduce metastasis in living organisms. Furthermore, vanillin has been shown to decrease cell growth in vitro, indicating its potential as an improved anti-metastatic drug. Notably, the anti-metastatic effect of vanillin was evident in living organisms at a well-tolerated dosage in mice [104].

Another significant compound identified in the extract was schizandrin, which is known for its anti-cancer properties, including its ability to operate as a dual inhibitor of P-glycoprotein and multidrug resistance protein 1 (MRP1), contributing to its efficacy against cancer cells [109]. Schizandrin also inhibits ATR protein kinase, specifically its response to DNA damage [110]. Schizandrin has been shown to produce a remarkable reduction in 4T1 lung metastasis and prolonged survival in mice. It largely inhibits 4T1 cell metastasis at the local invasion stage and reduces epithelial–mesenchymal transition (EMT) in both 4T1 and primary human breast cancer cells, lowering their metastatic potential [111]. The involvement of schizandrin in suppressing cancer cell metastasis, as well as its advantages when combined with other anti-cancer medications, highlights its diverse pharmacological effects, including antioxidant, anti-asthmatic, anti-inflammatory, and anti-cancer properties [21].

Schisandrin inhibits glioma cell growth and invasion by modulating several signaling pathways [112] and inhibits TGF-1-induced epithelial–mesenchymal transition (EMT) in human A549 cells [113]. Cell migration, invasion, epithelial–mesenchymal transition (EMT), and cancer stem cell (CSC) properties are inhibited by schisandrin. Furthermore, it may regulate additional signaling pathways linked with EMT, including the SMAD, PI3K/AKT, Wnt, and Notch pathways, indicating that schisandrin has a larger regulatory impact on these cancer-related processes. Schisandrin causes cell cycle arrest in A549 cells by downregulating cyclin D1, cyclin-dependent kinase (CDK) 4, and CDK6 while simultaneously upregulating p53 and p21 [114]. Schisandrin lowered SIRT1 protein expression, and there was a negative association between SIRT1 and the stimulation of SMURF2, which inhibits colon cancer cell proliferation and dissemination [115].

Taurolidine, another compound identified in the *T. violacea* extract, exhibits potent antineoplastic and cytotoxic activities, suggesting its potential as a chemotherapeutic agent [50]. Taurolidine exhibits anti-endotoxin, antimicrobial, anti-adhesive, and antifungal traits [116]. Taurolidine inhibited the growth of a rat metastatic colorectal tumor cell line in vitro and in vivo, indicating that it may be useful in preventing peritoneal metastases [117]. Taurolidine likely acts by directly diminishing IL-1 production in peritoneal macrophages, thereby obstructing the response of tumor cells to growth signals [118]. Consequently, taurolidine undergoes enzymatic hydrolysis and decomposes into methyloltaurultam and taurultam, which further breaks down into methyloltaurinamid, ultimately producing taurine and an active methylol group. However, the precise mechanism underlying the suppression of tumor growth by taurolidine in various cancers remains unclear [119]. In a Syrian hamster model of pancreatic adenocarcinoma, taurolidine effectively inhibited primary tumor growth and reduced metastases at both the chemotherapy port site and the liver [120]. The identification of a compound resembling the synthetic compound taurolidine implies that the *T. violacea* extract may contain one or more compounds that are natural product analogs with a similar anti-cancer activity. This possibility warrants further research in order to confirm the presence of these compounds and isolate them. Once isolated, their structural and functional similarity to taurolidine can be confirmed. These taurolidine-like compounds can then serve as lead compounds for the development of new therapeutic drugs to treat TNBC.

Alpha-pinene is a naturally occurring compound that exhibits anti-cancer characteristics [58]. Alpha-pinene was isolated from the water-soluble extract of *T. violacea*. Studies on human ovarian cancer cell lines and human hepatocellular liver carcinoma cell lines have shown that α-pinene has anti-cancer effects [52]. It has also been shown in tests on N2A neuroblastoma cells to have antioxidant, anti-cancer, and genotoxic effects [121]. The capacity of α-pinene to suppress tumor invasion was tested in a study employing highly metastatic MDA-MB-231 human breast cancer cells [122]. TNFα-induced matrix metalloproteinase-9 gene promoter activation and mRNA synthesis have been demonstrated to be inhibited by α-pinene in a dose-dependent manner [120]. NF-κB-dependent transcriptional activity was reduced by α-pinene treatment [123]. It can also suppress TNFα-induced MMP-9 gene expression and the invasive nature of MDA-MB-231 cells [124].

Finally, it is important to remember that when the extracts of *T. violacea* are used by traditional healers, they are using the entire extract and do not perform any purification methods that would isolate individual compounds. As such, the patient is treated with the entire mixture of compounds. We do not know what effect the compounds within the extract may have when working together as the complex mixture of compounds may result in certain compounds behaving very differently from how a pure version of that compound may behave. Perhaps it is the complex mixture of compounds which gives the extract its full ability and any attempt to make a treatment based on the extract should study the potential interactions between the compounds that make up the extract.

### 4.4. Transcript Analysis of Genes Involved in Migration, Invasion, and Adhesion

The analysis of the transcripts of genes involved in invasion, adhesion, and metastasis showed no clear pattern of changes in the pathways controlling these processes following treatment. Only a few genes in these pathways were altered after treatment with the water-soluble extract. Many genes showed insignificant changes in expression. The normal breast cell line had many genes whose transcripts were only detectable before or after treatment with the extract, which seemed to indicate that the extract induced greater changes in the normal cell line. The changes in the transcription levels of genes involved in migration, invasion, and adhesion did not reflect the changes in these processes that were observed following the treatment. Since the results of the assays seemed to indicate that the extract was able to inhibit migration and invasion while stimulating adhesion, we expected to observe a decrease in the level of transcription of genes involved in migration and invasion and an increase in the transcription of genes involved in adhesion.

The majority of previous studies on *T. violacea* extracts found anti-cancer activities in fractions obtained through the use of organic solvents. However, this study’s results agree with those of the study carried out by Saibu et al. [29], which showed activity for the water-soluble extract. All the previous studies on *T. violacea* extracts focused on the cytotoxic nature of the extract and showed that the extract could increase the levels of apoptosis by increasing the expression of p53 and caspase 3 while increasing the levels of ROS. The transcriptome data in this study showed similar increases in the expression of pro-apoptotic genes; however, the focus of this study was on the ability of the extract to prevent the spread of cancer cells by inhibiting cancer cell metastasis and invasion, as well as increasing the cell adhesion to an ECM. The identification of different active compounds in the water and organic solvent fractions is likely due to differences in the extraction methods. This study could have been improved with the use of a “stronger” non-polar solvent than methanol, as there may be many compounds with activity which remained unextracted as they could not be dissolved in the methanol or water.

## 5. Conclusions

The ability of the water-soluble extract to inhibit cell invasion and metastasis and promote cell adhesion in a TNBC cell line implies that this extract can decrease the spread and progression of triple-negative breast cancer. The water-soluble extract also has moderate antioxidant activity and can prevent oxidative damage and protect tumor cells from death induced by ROS. However, this is an effect of the extract as a whole, and future work should involve activity studies to isolate individual compounds that give the extract its cytotoxic, anti-metastatic, and antioxidative properties. This would identify the compounds that give this extract its desirable cytotoxic and anti-metastatic activities that could be used for TNBC treatment. Therefore, the extract has potential as a basis for the development of new anti-cancer therapies for TNBC.

These results showed that in addition to the cytotoxic effects of the extract on cancer cells, the water-soluble extract was able to prevent metastasis and invasion as well as promote adhesion in a TNBC cell line.

## Figures and Tables

**Table 1:** Some IC50 values for well-known antioxidants.

Compound	IC_50_	Ref(s).
Ascorbic acid	3.8 µg/mL	[34]
*Astragalus Alopecurus* var Maximus (Willd.)	115.5 µg/mL	[35]
Avocado *(Folium* perseae Mill.)	601 µg/mL	[36]
Caffeic acid	1.6 µg/mL	[37]
Cinnamon (*Cinnamomum verum* J. Presl)	21.3 µg/mL	[38]
Bindweed (*Convulvulus betonicifolia* Mill.)	346.5 µg/mL	[39]
Fennel (*Foeniculum vulgare* Mill.)	263.2 µg/mL	[40]
*Tulbaghia violacea* Harv.	393 μg/mL	This study
*Verbascum speciousum* Schrad.	173.3 µg/mL	[41]
Ginger (*Zingiber officinale* Roscoe)	16.2 µg/mL	[42]

**Table 2:** Examples of compounds detected by NMR analysis of crude water-soluble *T. violacea* extracts.

Phytochemical Compound	Exact Mass	Formula	Ref.
4H-Pyran-4-one,2,3-dihydro-3,5-dihydroxy-6-methyl-(DDMP)	144.12	C_6_H_8_O_4_	[43]
1,2,4-Triazine-3,5(2H,4H)-dione	113.08	C_3_H_3_N_3_O_2_	[44]
d-Glycero-d-galacto-heptose	210.18	C_7_H_14_O_7_	[45]
Benzaldehyde, 4-(1-methylethyl)	148.20	C_10_H_12_O	[46]
Vanillin	152.15	C_8_H_8_O_3_	[47]
Methoxy-phenyl oxime	151.16	C_8_H_9_NO_2_	[48]
Pyrrolidine	71.12	C_4_H_9_N	[49]
Schizandrin	432.50	C_24_H_32_O_7_	[50]
Taurolidine	284.40	C_7_H_16_N_4_O_4_S_2_	[51]
Alpha-pinene	136.23	C_10_H_16_	[52]
Terbutaline,N-trifluoroacetyl-o,o,o-tris(trimethylsilyl)	537.80	C_23_H_42_F_3_NO_4_Si_3_	[53]
Difenoxin	424.50	C_28_H_28_N_2_O_2_	[54]
Mephobarbital	246.26	C_13_H_14_N_2_O_3_	[55]
Benserazide	257.24	C_10_H_15_N_3_O_5_	[56]
Antipyrine	188.23	C_11_H_12_N_2_O	[57]
Tricyclo [3.3.1.1(3,7)] decan-1-amine	151.25	C_10_H_17_N	[58]
Thymol	150.22	C_10_H_14_O	[59]
Cyclandelate	276.40	C_17_H_24_O_3_	[60]
Benzene propanoic acid	150.17	C_9_H_10_O_2_	[61]
Ethchlorvynol	144.60	C_7_H_9_ClO	[62]
Cycloserine	102.09	C_3_H_6_N_2_O_2_	[63]
Emylcamate	145.20	C_7_H_15_NO_2_	[64]
2-Propen-1-amine	57.09	C_3_H_7_N	[65]
Methyl formate	60.05	C_2_H_4_O_2_	[66]
Tetradecamethyl-cycloheptasiloxane	519.07	C_14_H_42_O_7_Si_7_	[67]
Cyanogen chloride	61.47	CNCl	[68]

## Data Availability

The data presented in this study are available on request from the corresponding author.

## References

[1] Shin V.Y., Siu J.M., Cheuk I., Ng E.K., Kwong A. (2015). Circulating cell-free miRNAs as biomarker for triple-negative breast cancer. Br. J. Cancer.

[2] Dent R., Hanna W.M., Trudeau M., Rawlinson E., Sun P., Narod S.A. (2009). Pattern of metastatic spread in triple-negative breast cancer. Breast Cancer Res. Treat..

[3] Foulkes W.D., Smith I.E., Reis-Filho J.S. (2010). Triple-negative breast cancer. N. Engl. J. Med..

[4] Carey L.A., Dees E.C., Sawyer L., Gatti L., Moore D.T., Collichio F., Ollila D.W., Sartor C.I., Graham M.L., Perou C.M. (2007). The triple negative paradox: Primary tumor chemosensitivity of breast cancer subtypes. Clin. Cancer Res. Off. J. Am. Assoc. Cancer Res..

[5] Boyle P. (2012). Triple-negative breast cancer: Epidemiological considerations and recommendations. Ann. Oncol. Off. J. Eur. Soc. Med. Oncol..

[6] Lara-Medina F., Pérez-Sánchez V., Saavedra-Pérez D., Blake-Cerda M., Arce C., Motola-Kuba D., Villarreal-Garza C., González-Angulo A.M., Bargalló E., Aguilar J.L. (2011). Triple-negative breast cancer in Hispanic patients: High prevalence, poor prognosis, and association with menopausal status, body mass index, and parity. Cancer.

[7] Fostira F., Tsitlaidou M., Papadimitriou C., Pertesi M., Timotheadou E., Stavropoulou A.V., Glentis S., Bournakis E., Bobos M., Pectasides D. (2012). Prevalence of BRCA1 mutations among 403 women with triple-negative breast cancer: Implications for genetic screening selection criteria: A Hellenic Cooperative Oncology Group Study. Breast Cancer Res. Treat..

[8] Bamidele O., Ali N., Papadopoulos C., Randhawa G. (2017). Exploring factors contributing to low uptake of the NHS breast cancer screening programme among Black African women in the UK. Divers. Equal. Health Care.

[9] Newman L.A., Stark A., Chitale D., Pepe M., Longton G., Worsham M.J., Nathanson S.D., Miller P., Bensenhaver J.M., Proctor E. (2017). Association Between Benign Breast Disease in African American and White American Women and Subsequent Triple-Negative Breast Cancer. JAMA Oncol..

[10] Shah S.P., Roth A., Goya R., Oloumi A., Ha G., Zhao Y., Turashvili G., Ding J., Tse K., Haffari G. (2012). The clonal and mutational evolution spectrum of primary triple-negative breast cancers. Nature.

[11] Vagia E., Mahalingam D., Cristofanilli M. (2020). The Landscape of Targeted Therapies in TNBC. Cancers.

[12] Lee K.M., Giltnane J.M., Balko J.M., Schwarz L.J., Guerrero-Zotano A.L., Hutchinson K.E., Nixon M.J., Estrada M.V., Sánchez V., Sanders M.E. (2017). MYC and MCL1 Cooperatively Promote Chemotherapy-Resistant Breast Cancer Stem Cells via Regulation of Mitochondrial Oxidative Phosphorylation. Cell Metab..

[13] André F., Zielinski C.C. (2012). Optimal strategies for the treatment of metastatic triple-negative breast cancer with currently approved agents. Ann. Oncol. Off. J. Eur. Soc. Med. Oncol..

[14] Hayes D.F., Thor A.D., Dressler L.G., Weaver D., Edgerton S., Cowan D., Broadwater G., Goldstein L.J., Martino S., Ingle J.N. (2007). HER2 and response to paclitaxel in node-positive breast cancer. N. Engl. J. Med..

[15] Luo M., Zhou L., Huang Z., Li B., Nice E.C., Xu J., Huang C. (2022). Antioxidant Therapy in Cancer: Rationale and Progress. Antioxidants.

[16] Buchheit C.L., Weigel K.J., Schafer Z.T. (2014). Cancer cell survival during detachment from the ECM: Multiple barriers to tumour progression. Nat. Rev. Cancer.

[17] Schafer Z.T., Grassian A.R., Song L., Jiang Z., Gerhart-Hines Z., Irie H.Y., Gao S., Puigserver P., Brugge J.S. (2009). Antioxidant and oncogene rescue of metabolic defects caused by loss of matrix attachment. Nature.

[18] DeNicola G.M., Karreth F.A., Humpton T.J., Gopinathan A., Wei C., Frese K., Mangal D., Yu K.H., Yeo C.J., Calhoun E.S. (2011). Oncogene-induced Nrf2 transcription promotes ROS detoxification and tumorigenesis. Nature.

[19] Sayin V.I., Ibrahim M.X., Larsson E., Nilsson J.A., Lindahl P., Bergo M.O. (2014). Antioxidants accelerate lung cancer progression in mice. Sci. Transl. Med..

[20] Liu M.M., Huang Y., Wang J. (2012). Developing phytoestrogens for breast cancer prevention. Anti-Cancer Agents Med. Chem..

[21] Zhang Y., Liu X., Ruan J., Zhuang X., Zhang X., Li Z. (2020). Phytochemicals of garlic: Promising candidates for cancer therapy. Biomed. Pharmacother. = Biomed. Pharmacother..

[22] Newman D.J., Cragg G.M. (2012). Natural products as sources of new drugs over the 30 years from 1981 to 2010. J. Nat. Prod..

[23] Mans D.R., da Rocha A.B., Schwartsmann G. (2000). Anti-cancer drug discovery and development in Brazil: Targeted plant collection as a rational strategy to acquire candidate anti-cancer compounds. Oncologist.

[24] Yang B., Liu Z., Wang Q., Chai Y., Xia P. (2018). Pharmacokinetic comparison of seven major bioactive components in normal and depression model rats after oral administration of Baihe Zhimu decoction by liquid chromatography-tandem mass spectrometry. J. Pharm. Biomed. Anal..

[25] Slika H., Mansour H., Wehbe N., Nasser S.A., Iratni R., Nasrallah G., Shaito A., Ghaddar T., Kobeissy F., Eid A.H. (2022). Therapeutic potential of flavonoids in cancer: ROS-mediated mechanisms. Biomed. Pharmacother. = Biomed. Pharmacother..

[26] Van Wyk B.-E., Oudtshoorn B.v., Gericke N. (1997). Medicinal Plants of South Africa.

[27] Motadi L.R., Choene M.S., Mthembu N.N. (2020). Anticancer properties of *Tulbaghia violacea* regulate the expression of p53-dependent mechanisms in cancer cell lines. Sci. Rep..

[28] Takaidza S., Kumar A.M., Ssemakalu C.C., Natesh N.S., Karanam G., Pillay M. (2018). Anticancer activity of crude acetone and water extracts of *Tulbaghia violacea* on human oral cancer cells. Asian Pac. J. Trop. Biomed..

[29] Saibu G.M., Katerere D., Rees J., Meyer M. (2011). Evaluation of the anti-cancer phytotherapeutic potential of *Tulbaghia violacea* plant. FASEB J..

[30] Saibu G.M., Katerere D.R., Rees D.J., Meyer M. (2015). In vitro cytotoxic and pro-apoptotic effects of water extracts of *Tulbaghia violacea* leaves and bulbs. J. Ethnopharmacol..

[31] Boyden S. (1962). The chemotactic effect of mixtures of antibody and antigen on polymorphonuclear leucocytes. J. Exp. Med..

[32] Todaro G.J., Lazar G.K., Green H. (1965). The initiation of cell division in a contact-inhibited mammalian cell line. J. Cell. Physiol..

[33] Blois M. (1958). Antioxidant determinations by the use of a stable free radical. Nature.

[34] Sharma G., Sapkota B., Lamichhane G., Adhikari M., Kunwar P. (2017). Antioxidant Activity of Selected Medicinal Plants of Nepal. Int. J. Med. Biomed. Sci..

[35] Güven L., Erturk A., Miloğlu F.D., Alwasel S., Gulcin İ.J.P. (2023). Screening of antiglaucoma, antidiabetic, anti-alzheimer, and antioxidant activities of *Astragalus alopecurus* pall—Analysis of phenolics profiles by LC-MS/MS. Pharmaceuticals.

[36] Polat Kose L., Bingol Z., Kaya R., Goren A.C., Akincioglu H., Durmaz L., Koksal E., Alwasel S.H., Gülçin İ. (2020). Anticholinergic and antioxidant activities of avocado (*Folium perseae*) leaves–phytochemical content by LC-MS/MS analysis. Int. J. Food Prop..

[37] Cuvelier M.-E., Richard H., Berset C. (1992). Comparison of the antioxidative activity of some acid-phenols: Structure-activity relationship. Biosci. Biotechnol. Biochem..

[38] Gulcin I., Kaya R., Goren A.C., Akincioglu H., Topal M., Bingol Z., Cetin Çakmak K., Ozturk Sarikaya S.B., Durmaz L., Alwasel S. (2019). Anticholinergic, antidiabetic and antioxidant activities of cinnamon (*Cinnamomum verum*) bark extracts: Polyphenol contents analysis by LC-MS/MS. Int. J. Food Prop..

[39] Bingol Z., Kızıltaş H., Gören A.C., Kose L.P., Topal M., Durmaz L., Alwasel S.H., Gulcin I.J.H. (2021). Antidiabetic, anticholinergic and antioxidant activities of aerial parts of shaggy bindweed (*Convulvulus betonicifolia* Miller subsp.)—Profiling of phenolic compounds by LC-HRMS. Heliyon.

[40] Oktay M., Gülçin İ., Küfrevioğlu Ö.İ. (2003). Determination of in vitro antioxidant activity of fennel (*Foeniculum vulgare*) seed extracts. LWT-Food Sci. Technol..

[41] Kızıltas H., Bingol Z., Goren A., Alwasel S., Gulcin I. (2023). Verbascum speciousum Schrad: Analysis of phenolic compounds by LC-HRMS and determination of antioxidant and enzyme inhibitory properties. Rec. Nat. Prod.

[42] Tohma H., Gülçin İ., Bursal E., Gören A.C., Alwasel S.H., Köksal E. (2017). Antioxidant activity and phenolic compounds of ginger (*Zingiber officinale* Rosc.) determined by HPLC-MS/MS. J. Food Meas. Charact..

[43] Ban J.O., Hwang I.G., Kim T.M., Hwang B.Y., Lee U.S., Jeong H.S., Yoon Y.W., Kimz D.J., Hong J.T. (2007). Anti-proliferate and pro-apoptotic effects of 2,3-dihydro-3,5-dihydroxy-6-methyl-4H-pyranone through inactivation of NF-kappaB in human colon cancer cells. Arch. Pharmacal Res..

[44] Sorm F., Jakubovic A., Slechta L. (1956). The anticancerous action of 6-azauracil (3,5-dioxo-2,3,4,5-tetrahydro-1,2,4-triazine). Experientia.

[45] Liang L., Wade Wei T.Y., Wu P.Y., Herrebout W., Tsai M.D., Vincent S.P. (2020). Nonhydrolyzable Heptose Bis- and Monophosphate Analogues Modulate Pro-inflammatory TIFA-NF-κB Signaling. Chembiochem. Eur. J. Chem. Biol..

[46] Tsai K.D., Liu Y.H., Chen T.W., Yang S.M., Wong H.Y., Cherng J., Chou K.S., Cherng J.M. (2016). Cuminaldehyde from Cinnamomum verum Induces Cell Death through Targeting Topoisomerase 1 and 2 in Human Colorectal Adenocarcinoma COLO 205 Cells. Nutrients.

[47] Yan Y.Q., Xu Q.Z., Wang L., Sui J.L., Bai B., Zhou P.K. (2006). Vanillin derivative 6-bromine-5-hydroxy-4-methoxybenzaldehyde-elicited apoptosis and G2/M arrest of Jurkat cells proceeds concurrently with DNA-PKcs cleavage and Akt inactivation. Int. J. Oncol..

[48] Ozma M.A., Ghotaslou R., Asgharzadeh M., Abbasi A., Rezaee M.A., Kafil H.S. (2024). Cytotoxicity assessment and antimicrobial effects of cell-free supernatants from probiotic lactic acid bacteria and yeast against multi-drug resistant *Escherichia coli*. Lett. Appl. Microbiol..

[49] Sajjad F., You Q., Xing D., Fan H., Reddy A.G.K., Hu W., Dong S. (2020). Synthesis and biological evaluation of substituted pyrrolidines and pyrroles as potential anticancer agents. Arch. Pharm..

[50] Lv X.J., Zhao L.J., Hao Y.Q., Su Z.Z., Li J.Y., Du Y.W., Zhang J. (2015). Schisandrin B inhibits the proliferation of human lung adenocarcinoma A549 cells by inducing cycle arrest and apoptosis. Int. J. Clin. Exp. Med..

[51] Baker D.M., Jones J.A., Nguyen-Van-Tam J.S., Lloyd J.H., Morris D.L., Bourke J.B., Steele R.J., Hardcastle J.D. (1994). Taurolidine peritoneal lavage as prophylaxis against infection after elective colorectal surgery. Br. J. Surg..

[52] Aydin E., Türkez H., Geyikoğlu F. (2013). Antioxidative, anticancer and genotoxic properties of α-pinene on N2a neuroblastoma cells. Biologia.

[53] Duan X., Yang Y., Yang A., Zhao Y., Fan F., Niu L., Hao N. (2022). Terbutaline attenuates LPS-induced injury of pulmonary microvascular endothelial cells by cAMP/Epac signaling. Drug Dev. Res..

[54] Paul A. (2021). Antidiarrheal agents. Introduction to Basics of Pharmacology Toxicology: Volume 2: Essentials of Systemic Pharmacology: From Principles to Practice.

[55] Matin M.A., Jaffery F.N., Kar P.P. (1980). Role of striatal acetylcholine and free ammmonia in the central stimulatory effects of pp’DDT in rats. Protective effects of barbiturates. Arch. Toxicol..

[56] Ryan M., Slevin J.T. (2006). Restless legs syndrome. Am. J. Health-Syst. Pharm..

[57] Refat M.S., Hamza R.Z., Adam A., Saad H.A., Gobouri A.A., Al-Salmi F.A., Altalhi T., El-Megharbel S.M. (2021). Synthesis of N,N′-bis(1,5-dimethyl-2-phenyl-1,2-dihydro-3-oxopyrazol-4-yl) sebacamide that ameliorate osteoarthritis symptoms and improve bone marrow matrix structure and cartilage alterations induced by monoiodoacetate in the rat model: “Suggested potent anti-inflammatory agent against COVID-19”. Hum. Exp. Toxicol..

[58] Parkes J. (1989). Clinical pharmacology of amantadine and derivatives. Early Diagnosis and Preventive Therapy in Parkinson’s Disease.

[59] Hammoudi Halat D., Krayem M., Khaled S., Younes S. (2022). A Focused Insight into Thyme: Biological, Chemical, and Therapeutic Properties of an Indigenous Mediterranean Herb. Nutrients.

[60] Ma Y.-Z., Qiang G.-F., Du G.-H., Du G.-H. (2018). Cyclandelate. Natural Small Molecule Drugs from Plants.

[61] Bu R., Xie J., Yu J., Liao W., Xiao X., Lv J., Wang C., Ye J., Calderón-Urrea A. (2016). Autotoxicity in cucumber (*Cucumis sativus* L.) seedlings is alleviated by silicon through an increase in the activity of antioxidant enzymes and by mitigating lipid peroxidation. J. Plant Biol..

[62] Parker F.S., Parker F.S. (1971). Drugs, Pharmaceuticals, and Pharmacological Applications. Applications of Infrared Spectroscopy in Biochemistry, Biology, and Medicine.

[63] van der Galiën R., Boveneind-Vrubleuskaya N.V., Peloquin C., Skrahina A., Touw D.J., Alffenaar J.C. (2020). Pharmacokinetic Modeling, Simulation, and Development of a Limited Sampling Strategy of Cycloserine in Patients with Multidrug-/Extensively Drug-Resistant Tuberculosis. Clin. Pharmacokinet..

[64] Smith J.M., Misiak H. (1976). Critical flicker frequency (CFF) and psychotropic drugs in normal human subjects-a review. Psychopharmacologia.

[65] Arken N. (2019). Schizandrol A reverses multidrug resistance in resistant chronic myeloid leukemia cells K562/A02. Cell. Mol. Biol..

[66] Stedjan M.K., Augspurger J.D. (2015). Ring strain energy in ether-and lactone-containing spiro compounds. J. Phys. Org. Chem..

[67] Omoruyi B.E., Afolayan A.J., Bradley G. (2014). The inhibitory effect of *Mesembryanthemum edule* (L.) bolus essential oil on some pathogenic fungal isolates. BMC Complement. Altern. Med..

[68] Zheng A., Dzombak D.A., Luthy R.G. (2004). Formation of free cyanide and cyanogen chloride from chloramination of publicly owned treatment works secondary effluent: Laboratory study with model compounds. Water Environ. Res. Res. Publ. Water Environ. Fed..

[69] Neal C.L., McKeithen D., Odero-Marah V.A. (2011). Snail negatively regulates cell adhesion to extracellular matrix and integrin expression via the MAPK pathway in prostate cancer cells. Cell Adhes. Migr..

[70] Chen Q., Xu R., Zeng C., Lu Q., Huang D., Shi C., Zhang W., Deng L., Yan R., Rao H. (2014). Down-regulation of Gli transcription factor leads to the inhibition of migration and invasion of ovarian cancer cells via integrin β4-mediated FAK signaling. PLoS ONE.

[71] Chen J.S., Huang X.H., Wang Q., Huang J.Q., Zhang L.J., Chen X.L., Lei J., Cheng Z.X. (2013). Sonic hedgehog signaling pathway induces cell migration and invasion through focal adhesion kinase/AKT signaling-mediated activation of matrix metalloproteinase (MMP)-2 and MMP-9 in liver cancer. Carcinogenesis.

[72] Aravani D., Morris G.E., Jones P.D., Tattersall H.K., Karamanavi E., Kaiser M.A., Kostogrys R.B., Ghaderi Najafabadi M., Andrews S.L., Nath M. (2019). HHIPL1, a Gene at the 14q32 Coronary Artery Disease Locus, Positively Regulates Hedgehog Signaling and Promotes Atherosclerosis. Circulation.

[73] Drosten M., Dhawahir A., Sum E.Y., Urosevic J., Lechuga C.G., Esteban L.M., Castellano E., Guerra C., Santos E., Barbacid M. (2010). Genetic analysis of Ras signalling pathways in cell proliferation, migration and survival. EMBO J..

[74] Huang C., Jacobson K., Schaller M.D. (2004). MAP kinases and cell migration. J. Cell Sci..

[75] Singh S.P., Paschke P., Tweedy L., Insall R.H. (2022). AKT and SGK kinases regulate cell migration by altering Scar/WAVE complex activation and Arp2/3 complex recruitment. Front. Mol. Biosci..

[76] Wang Q., Liu H., Wang Q., Zhou F., Liu Y., Zhang Y., Ding H., Yuan M., Li F., Chen Y. (2017). Involvement of c-Fos in cell proliferation, migration, and invasion in osteosarcoma cells accompanied by altered expression of Wnt2 and Fzd9. PLoS ONE.

[77] Dorudi S., Hart I.R. (1993). Mechanisms underlying invasion and metastasis. Curr. Opin. Oncol..

[78] Gupta G.P., Massagué J. (2006). Cancer metastasis: Building a framework. Cell.

[79] Huang S., Ingber D.E. (1999). The structural and mechanical complexity of cell-growth control. Nat. Cell Biol..

[80] Okegawa T., Pong R.C., Li Y., Hsieh J.T. (2004). The role of cell adhesion molecule in cancer progression and its application in cancer therapy. Acta Biochim. Pol..

[81] Hirohashi S., Kanai Y. (2003). Cell adhesion system and human cancer morphogenesis. Cancer Sci..

[82] Omar A., Jovanovic K., Da Costa Dias B., Gonsalves D., Moodley K., Caveney R., Mbazima V., Weiss S.F. (2011). Patented biological approaches for the therapeutic modulation of the 37 kDa/67 kDa laminin receptor. Expert Opin. Ther. Pat..

[83] Akula U.S., Odhav B. (2008). In vitro 5-Lipoxygenase inhibition of polyphenolic antioxidants from undomesticated plants of South Africa. J. Med. Plants Res..

[84] Hsu C.Y., Chan Y.P., Chang J. (2007). Antioxidant activity of extract from Polygonum cuspidatum. Biol. Res..

[85] Olorunnisola O.S., Bradley G., Afolayan A.J. (2012). Protective effect of *T. violacea* rhizome extract against hypercholesterolemia-induced oxidative stress in Wistar rats. Molecules.

[86] Madike L.N., Takaidza S., Ssemakalu C., Pillay M. (2019). Genotoxicity of aqueous extracts of *Tulbaghia violacea* as determined through an Allium cepa assay. S. Afr. J. Sci..

[87] Naidoo V., McGaw L.J., Bisschop S.P., Duncan N., Eloff J.N. (2008). The value of plant extracts with antioxidant activity in attenuating coccidiosis in broiler chickens. Vet. Parasitol..

[88] Ncube B., Finnie J.F., Van Staden J. (2012). In vitro antimicrobial synergism within plant extract combinations from three South African medicinal bulbs. J. Ethnopharmacol..

[89] Takara K., Otsuka K., Wada K., Iwasaki H., Yamashita M. (2007). 1,1-Diphenyl-2-picrylhydrazyl radical scavenging activity and tyrosinase inhibitory effects of constituents of sugarcane molasses. Biosci. Biotechnol. Biochem..

[90] Yu X., Zhao M., Liu F., Zeng S., Hu J. (2013). Identification of 2,3-dihydro-3, 5-dihydroxy-6-methyl-4H-pyran-4-one as a strong antioxidant in glucose–histidine Maillard reaction products. Food Res. Int..

[91] Mills F., Weisleder D., Hodge J. (1970). 2,3-Dihydro-3,5-dihydroxy-6-methyl-4H-pyran-4-one, a novel nonenzymatic browning product. Tetrahedron Lett..

[92] Ibrahim D., Abdelfattah-Hassan A., Badawi M., Ismail T.A., Bendary M.M., Abdelaziz A.M., Mosbah R.A., Mohamed D.I., Arisha A.H., El-Hamid M.I.A. (2021). Thymol nanoemulsion promoted broiler chicken’s growth, gastrointestinal barrier and bacterial community and conferred protection against Salmonella Typhimurium. Sci. Rep..

[93] Magi S., Tashiro E., Imoto M. (2012). A chemical genomic study identifying diversity in cell migration signaling in cancer cells. Sci. Rep..

[94] Todaro M., Gaggianesi M., Catalano V., Benfante A., Iovino F., Biffoni M., Apuzzo T., Sperduti I., Volpe S., Cocorullo G. (2014). CD44v6 is a marker of constitutive and reprogrammed cancer stem cells driving. Cell Stem Cell.

[95] Hin N., Duvall B., Ferraris D., Alt J., Thomas A.G., Rais R., Rojas C., Wu Y., Wozniak K.M., Slusher B.S. (2015). 6-Hydroxy-1,2,4-triazine-3,5(2H,4H)-dione Derivatives as Novel D-Amino Acid Oxidase Inhibitors. J. Med. Chem..

[96] John V., Raju G.P., Lakshmi G., Rani B.L., Bollikolla H.B. (2020). Developments on 1,2,4-triazine scaffold substitutions for possible anticancer agents. Caribb. J. Sci..

[97] Harris A., Grahame-Smith D., Potter C., Bunch C. (1981). Cytosine arabinoside deamination in human leukaemic myeloblasts and resistance to cytosine arabinoside therapy. Clin. Sci..

[98] Krynetski E.Y., Schuetz J.D., Galpin A.J., Pui C.H., Relling M.V., Evans W.E. (1995). A single point mutation leading to loss of catalytic activity in human thiopurine S-methyltransferase. Proc. Natl. Acad. Sci. USA.

[99] He S., Zhang C., Shafi A.A., Sequeira M., Acquaviva J., Friedland J.C., Sang J., Smith D.L., Weigel N.L., Wada Y. (2013). Potent activity of the Hsp90 inhibitor ganetespib in prostate cancer cells irrespective of androgen receptor status or variant receptor expression. Int. J. Oncol..

[100] Hernández D., Boto A. (2014). Nucleoside analogues: Synthesis and biological properties of azanucleoside derivatives. Eur. J. Org. Chem..

[101] Abdelnabi R., Delang L. (2020). Antiviral Strategies against Arthritogenic Alphaviruses. Microorganisms.

[102] Sankar M., Nowicka E., Carter E., Murphy D.M., Knight D.W., Bethell D., Hutchings G.J. (2014). The benzaldehyde oxidation paradox explained by the interception of peroxy radical by benzyl alcohol. Nat. Commun..

[103] Ahmed W., Sala C., Hegde S.R., Jha R.K., Cole S.T., Nagaraja V. (2017). Transcription facilitated genome-wide recruitment of topoisomerase I and DNA gyrase. PLoS Genet..

[104] Bezerra D.P., Soares A.K., de Sousa D.P. (2016). Overview of the Role of Vanillin on Redox Status and Cancer Development. Oxidative Med. Cell. Longev..

[105] Ho K., Yazan L.S., Ismail N., Ismail M. (2011). Toxicology study of vanillin on rats via oral and intra-peritoneal administration. Food Chem. Toxicol. Int. J. Publ. Br. Ind. Biol. Res. Assoc..

[106] Liang J.A., Wu S.L., Lo H.Y., Hsiang C.Y., Ho T.Y. (2009). Vanillin inhibits matrix metalloproteinase-9 expression through down-regulation of nuclear factor-kappaB signaling pathway in human hepatocellular carcinoma cells. Mol. Pharmacol..

[107] Gallage N.J., Hansen E.H., Kannangara R., Olsen C.E., Motawia M.S., Jørgensen K., Holme I., Hebelstrup K., Grisoni M., Møller B.L. (2014). Vanillin formation from ferulic acid in Vanilla planifolia is catalysed by a single enzyme. Nat. Commun..

[108] Marton A., Kúsz E., Kolozsi C., Tubak V., Zagotto G., Buzás K., Quintieri L., Vizler C. (2016). Vanillin Analogues o-Vanillin and 2,4,6-Trihydroxybenzaldehyde Inhibit NFĸB Activation and Suppress Growth of A375 Human Melanoma. Anticancer Res..

[109] Panossian A., Wikman G. (2008). Pharmacology of Schisandra chinensis Bail.: An overview of Russian research and uses in medicine. J. Ethnopharmacol..

[110] Hammond E.M., Denko N.C., Dorie M.J., Abraham R.T., Giaccia A.J. (2002). Hypoxia links ATR and p53 through replication arrest. Mol. Cell. Biol..

[111] Zhang Y., Weinberg R.A. (2018). Epithelial-to-mesenchymal transition in cancer: Complexity and opportunities. Front. Med..

[112] Lin X., Attar R., Mobeen I., Yulaevna I.M., Aras A., Butt G., Farooqi A.A. (2021). Regulation of cell signaling pathways by Schisandrin in different cancers: Opting for “Swiss Army Knife” instead of “Blunderbuss”. Cell. Mol. Biol..

[113] Zhuang W., Li Z., Dong X., Zhao N., Liu Y., Wang C., Chen J. (2019). Schisandrin B inhibits TGF-β1-induced epithelial-mesenchymal transition in human A549 cells through epigenetic silencing of ZEB1. Exp. Lung Res..

[114] Zhang Z., Guo S., Liu X., Gao X. (2015). Synergistic antitumor effect of α-pinene and β-pinene with paclitaxel against non-small-cell lung carcinoma (NSCLC). Drug Res..

[115] Pu Z., Zhang W., Wang M., Xu M., Xie H., Zhao J. (2021). Schisandrin B Attenuates Colitis-Associated Colorectal Cancer through SIRT1 Linked SMURF2 Signaling. Am. J. Chin. Med..

[116] Vernon-Roberts A., Lopez R.N., Frampton C.M., Day A.S. (2022). Meta-analysis of the efficacy of taurolidine in reducing catheter-related bloodstream infections for patients receiving parenteral nutrition. JPEN J. Parenter. Enter. Nutr..

[117] McCourt M., Wang J.H., Sookhai S., Redmond H.P. (2000). Taurolidine inhibits tumor cell growth in vitro and in vivo. Ann. Surg. Oncol..

[118] Wouters Y., Mennen G.R.H., Te Morsche R.H.M., Roelofs H.M.J., Wanten G.J.A. (2022). The Antiseptic and Antineoplastic Agent Taurolidine Modulates Key Leukocyte Functions. In Vivo.

[119] Ogura T., Tanaka Y., Tamaki H., Harada M. (2016). Docetaxel induces Bcl-2- and pro-apoptotic caspase-independent death of human prostate cancer DU145 cells. Int. J. Oncol..

[120] Wenger F.A., Kilian M., Braumann C., Neumann A., Ridders J., Peter F.J., Guski H., Jacobi C.A. (2002). Effects of taurolidine and octreotide on port site and liver metastasis after laparoscopy in an animal model of pancreatic cancer. Clin. Exp. Metastasis.

[121] Abe M., Asada N., Kimura M., Fukui C., Yamada D., Wang Z., Miyake M., Takarada T., Ono M., Aoe M. (2024). Antitumor activity of α-pinene in T-cell tumors. Cancer Sci..

[122] Salehi B., Upadhyay S., Erdogan Orhan I., Kumar Jugran A., L.D. Jayaweera S., A. Dias D., Sharopov F., Taheri Y., Martins N., Baghalpour N. (2019). Therapeutic Potential of α- and β-Pinene: A Miracle Gift of Nature. Biomolecules.

[123] Matsuda T., Shimada M., Sato A., Akase T., Yoshinari K., Nagata K., Yamazoe Y. (2011). Tumor necrosis factor-alpha-nuclear factor-kappa B-signaling enhances St2b2 expression during 12-O-tetradecanoylphorbol-13-acetate-induced epidermal hyperplasia. Biol. Pharm. Bull..

[124] Neves A., Rosa S., Gonçalves J., Rufino A., Judas F., Salgueiro L., Lopes M.C., Cavaleiro C., Mendes A.F. (2010). Screening of five essential oils for identification of potential inhibitors of IL-1-induced Nf-kappaB activation and NO production in human chondrocytes: Characterization of the inhibitory activity of alpha-pinene. Planta Medica.

